# Supplementary material for: Impact of COVID-19 outbreak on the mental health status of undergraduate medical students in a COVID-19 treating medical college: a prospective longitudinal study
Source: PeerJ. 2020 Oct 16;8:e10164. doi: 10.7717/peerj.10164 (PMC7571415; doi:10.7717/peerj.10164)
Supplement: Supplemental Information 1 — Extraction Method: Principal Component Analysis. Rotation Method: Varimax with Kaiser Normalization.a a. Rotation converged in 3 iterations. [file peerj-08-10164-s001.docx]

Supplementary table 1: Exploratory factor analysis of the self-administered questionnaire (Items 4 to 12)

| **Rotated Component Matrix^a^** | | |
| --- | --- | --- |
|  | Component | |
|  | 1 | 2 |
| 4. How likely are you to contract COVID-19? | .910 |  |
| 5. I worry about surviving, if contracted with COVID-19 | .910 |  |
| 6. I worry that I have been infected with COVID-19 | .875 |  |
| 7. I worry about the poor relationship between family members, friends and me because of COVID-19 pandemic | .875 |  |
| 8. I am concerned about my family members contracting COVID-19 | .621 |  |
| 9. I worry that the COVID-19 outbreak hinders with my acquisition of theoretical knowledge (Lecture sessions) |  | .932 |
| 10. I worry that the COVID-19 outbreak affects my acquisition of practical/ clinical skills |  | .927 |
| 11. I worry that the COVID-19 outbreak would affect my grades in the end of year examination |  | .879 |
| 12. I worry that the COVID-19 outbreak would affect my future prospects in medical carrier |  | .833 |

Extraction Method: Principal Component Analysis.

Rotation Method: Varimax with Kaiser Normalization.^a^

a. Rotation converged in 3 iterations
