# Supplementary material for: Impact of COVID-19 outbreak on the mental health status of undergraduate medical students in a COVID-19 treating medical college: a prospective longitudinal study
Source: PeerJ. 2020 Oct 16;8:e10164. doi: 10.7717/peerj.10164 (PMC7571415; doi:10.7717/peerj.10164)
Supplement: Supplemental Information 2 — OR Odds ratio; 95% CI 95% confidence interval [file peerj-08-10164-s002.docx]

Supplementary table S2: Binary logistic regression for baseline and follow-up scores of depression with Sociodemographic variables as independent variables.

| Variables | Sub-Categories | Baseline | | | | Follow-up | | | |
| --- | --- | --- | --- | --- | --- | --- | --- | --- | --- |
|  |  | OR | 95% CI | | P Value | OR | 95% CI | | P Value |
|  |  |  | Lower | Upper |  |  | Lower | Upper |  |
| Gender | Male* |  |  |  | - |  |  |  | - |
|  | Female | 1.746 | 0.931 | 3.271 | 0.082 | 0.945 | 0.522 | 1.709 | 0.851 |
| Age | Age | 0.887 | 0.687 | 1.143 | 0.354 | 0.737 | 0.565 | 0.961 | **0.024** |
| Current residence | Urban* |  |  |  | - |  |  |  | - |
|  | Rural | 1.184 | 0.631 | 2.223 | 0.598 | 1.172 | 0.631 | 2.180 | 0.615 |
| Year of study | Pre/paraclinical* |  |  |  | - |  |  |  | - |
|  | Clinical | 1.113 | 0.469 | 2.641 | 0.808 | 2.237 | 0.950 | 5.266 | 0.065 |
| Family Income | More than 1,00,000 INR* |  |  |  | 0.051 |  |  |  | 0.589 |
|  | Less than 50,000 INR | 2.510 | 1.153 | 5.461 | **0.020** | 1.472 | 0.698 | 3.107 | 0.310 |
|  | 50,000- 1,00,000 INR | 1.361 | 0.640 | 2.893 | 0.423 | 1.181 | 0.575 | 2.425 | 0.650 |

OR Odds ratio; 95% CI 95% confidence interval
