# Supplementary material for: Impact of COVID-19 outbreak on the mental health status of undergraduate medical students in a COVID-19 treating medical college: a prospective longitudinal study
Source: PeerJ. 2020 Oct 16;8:e10164. doi: 10.7717/peerj.10164 (PMC7571415; doi:10.7717/peerj.10164)
Supplement: Supplemental Information 3 — OR Odds ratio; 95% CI 95% confidence interval [file peerj-08-10164-s003.docx]

Supplementary table S3: Binary logistic regression for baseline and follow-up scores of anxiety with Sociodemographic variables as independent variables.

| Variables | Sub-Categories | Baseline | | | | Follow-up | | | |
| --- | --- | --- | --- | --- | --- | --- | --- | --- | --- |
|  |  | OR | 95% CI | | P Value | OR | 95% CI | | P Value |
|  |  |  | Lower | Upper |  |  | Lower | Upper |  |
| Gender | Male* |  |  |  | - |  |  |  | - |
|  | Female | 0.680 | 0.345 | 1.340 | 0.265 | 0.928 | 0.513 | 1.680 | 0.805 |
| Age | Age | 0.921 | 0.686 | 1.237 | 0.585 | 0.975 | 0.769 | 1.235 | 0.831 |
| Current residence | Urban* |  |  |  | - |  |  |  | - |
|  | Rural | 1.304 | 0.638 | 2.667 | 0.467 | 1.050 | 0.563 | 1.957 | 0.878 |
| Year of study | Pre/paraclinical* |  |  |  | - |  |  |  | - |
|  | Clinical | 0.754 | 0.277 | 2.051 | 0.580 | 1.098 | 0.482 | 2.503 | 0.824 |
| Family Income | More than 1,00,000 INR* |  |  |  | 0.309 |  |  |  | 0.827 |
|  | Less than 50,000 INR | 2.031 | 0.820 | 5.034 | 0.126 | 1.131 | 0.540 | 2.370 | 0.744 |
|  | 50,000- 1,00,000 INR | 1.593 | 0.656 | 3.868 | 0.304 | 0.914 | 0.449 | 1.862 | 0.805 |

OR Odds ratio; 95% CI 95% confidence interval
