# Supplementary material for: Impact of COVID-19 outbreak on the mental health status of undergraduate medical students in a COVID-19 treating medical college: a prospective longitudinal study
Source: PeerJ. 2020 Oct 16;8:e10164. doi: 10.7717/peerj.10164 (PMC7571415; doi:10.7717/peerj.10164)
Supplement: Supplemental Information 4 — OR Odds ratio; 95% CI 95% confidence interval [file peerj-08-10164-s004.docx]

Supplementary table S4: Binary logistic regression for baseline and follow-up scores of stress with Sociodemographic variables as independent variables.

| Variables | Sub-Categories | Baseline | | | | Follow-up | | | |
| --- | --- | --- | --- | --- | --- | --- | --- | --- | --- |
|  |  | OR | 95% CI | | P Value | OR | 95% CI | | P Value |
|  |  |  | Lower | Upper |  |  | Lower | Upper |  |
| Gender | Male* |  |  |  | - |  |  |  | - |
|  | Female | 0.833 | 0.420 | 1.652 | 0.600 | 0.735 | 0.386 | 1.399 | 0.348 |
| Age | Age | 0.894 | 0.668 | 1.196 | 0.450 | 0.986 | 0.760 | 1.280 | 0.916 |
| Current residence | Urban* |  |  |  | - |  |  |  | - |
|  | Rural | 1.470 | 0.722 | 2.992 | 0.288 | 1.296 | 0.659 | 2.548 | 0.452 |
| Year of study | Preclinical* |  |  |  | - |  |  |  | - |
|  | Clinical | 1.280 | 0.486 | 3.369 | 0.618 | 1.162 | 0.474 | 2.846 | 0.742 |
| Family Income | More than 1,00,000 INR* |  |  |  | 0.655 |  |  |  | 0.546 |
|  | Less than 50,000 INR | 1.240 | 0.529 | 2.909 | 0.621 | 1.372 | 0.625 | 3.010 | 0.431 |
|  | 50,000- 1,00,000 INR | 0.859 | 0.366 | 2.013 | 0.726 | 0.645 | 0.285 | 1.456 | 0.291 |

OR Odds ratio; 95% CI 95% confidence interval
