# Supplementary material for: Impact of COVID-19 outbreak on the mental health status of undergraduate medical students in a COVID-19 treating medical college: a prospective longitudinal study
Source: PeerJ. 2020 Oct 16;8:e10164. doi: 10.7717/peerj.10164 (PMC7571415; doi:10.7717/peerj.10164)
Supplement: Supplemental Information 6 [file peerj-08-10164-s006.docx]

**Survey questionnaire**

Choose only one option

1. Have you been tested for COVID-19? Yes / No

If yes, was the result positive/negative?

2. Has any of your family and/or friends tested positive for COVID-19?

Yes / No / I don’t know

3. Do you have any direct interactions with COVID-19 positive patients?

Yes / No / I don’t know

State your response on a scale of 1 to 5 (*With 1 being lowest and 5 being the maximum*)

4. How likely are you to contract COVID-19?

| 1 | 2 | 3 | 4 | 5 |
| --- | --- | --- | --- | --- |

5. I worry about surviving, if contracted with COVID-19

| 1 | 2 | 3 | 4 | 5 |
| --- | --- | --- | --- | --- |

6. I worry that I have been infected with COVID-19

| 1 | 2 | 3 | 4 | 5 |
| --- | --- | --- | --- | --- |

7. I worry about the poor relationship between family members, friends and me because of COVID-19 pandemic

| 1 | 2 | 3 | 4 | 5 |
| --- | --- | --- | --- | --- |

8. I am concerned about my family members contracting COVID-19

| 1 | 2 | 3 | 4 | 5 |
| --- | --- | --- | --- | --- |

9. I worry that the COVID-19 outbreak hinders with my acquisition of theoretical knowledge

| 1 | 2 | 3 | 4 | 5 |
| --- | --- | --- | --- | --- |

10. I worry that the COVID-19 outbreak affects my acquisition of practical / clinical skills

| 1 | 2 | 3 | 4 | 5 |
| --- | --- | --- | --- | --- |

| 1 | 2 | 3 | 4 | 5 |
| --- | --- | --- | --- | --- |

11. I worry that the COVID-19 outbreak would affect my grades in the end of year examination

12. I worry that the COVID-19 outbreak would affect my future prospects in medical carrier

| 1 | 2 | 3 | 4 | 5 |
| --- | --- | --- | --- | --- |
